# Existential Approaches and Cognitive Behavior Therapy: Challenges and Potential

**DOI:** 10.1007/s41811-020-00096-1

**Published:** 2021-01-04

**Authors:** Thomas Heidenreich, Alexander Noyon, Michael Worrell, Ross Menzies

**Affiliations:** 1 Social Work, Health and Care, University of Applied Sciences Esslingen, Flandernstr. 101, 73728 Esslingen am Neckar, Germany; 2 University of Applied Sciences Mannheim, Mannheim, Germany; 3 Central and North West London Foundation NHS Trust, London, UK; 4 University of Technology Sydney, Ultimo, Australia

**Keywords:** Existential concerns, Death, CBT, Values, Responsibility, Supervision, Therapy

## Abstract

Existential concerns such as death, responsibility, meaninglessness, and isolation not only are the hallmark of existential psychotherapy but also are frequently encountered by CBT therapists—nevertheless, due to epistemological and ideological differences, existential and CBT approaches to psychotherapy had little overlap historically. During recent years, existential issues are increasingly discussed in empirical clinical psychology, e.g., the potential role of the fear of death for a variety of mental disorders by Iverach et al. (*Clinical Psychology Review*, 34(7), 580–593, 2014), and there is increasing experimental evidence for a causal rather than correlational role of death anxiety discussed by Menzies and Dar-Nimrod (*Journal of Abnormal Psychology*, 126(4), 367–377, 2017). Further, existential concerns are common themes in CBT discussed by Grober et al. (*Psychotherapeut*, 61(3), 229–236, 2016) and may play an important role in the training of CBT therapists discussed by Worrell et al. (*Journal of Psychotherapy and Counselling Psychology Reflections*, 3(1), 9–16, 2018) as well as in personal therapy and supervision.

## Introduction: Existential Approaches to Psychotherapy and Their Relation to CBT

This paper is the result of an exchange between the four authors that took place during a panel discussion at the 9th World Congress of Cognitive and Behavioral Therapies (WCBCT) in Berlin in July 2019. The congress theme “CBT at the crossroads” allowed the authors to explore the potential of CBT’s encounters with existential themes and therapies as well as define the challenges of this encounter more clearly. While all four authors have a background in CBT, they share a long-term interest—and some are also formally trained—in existential psychotherapy. The role of existential concerns in psychotherapy and, notably, the relationship between CBT and existential issues was the main theme of the panel discussion that also focused on issues such as the role of death anxiety in mental disorders (and its implications for CBT) and the potential role of existential concerns for CBT training and supervision.

Following the exchange in the WCBCT panel discussion, we will explore some of the issues in more detail. Starting with the role of existential concerns (death, freedom, meaninglessness, and isolation) as formulated by Yalom (1980) and a general outline of existential phenomenology (Spinelli 2015), we will explore the potential of death as an existential approach for the understanding and treatment of psychopathology and will further explore the potential role of existential phenomenology for the development of therapists as well as supervision.

### Epistemological Background of Existential and CBT Approaches

Cognitive behavioral and existential therapies developed independently and, in spite of a potential comparability that will be explored in this paper, are often critical of each other: while CBT traces its roots to a behavioral tradition that aims at explaining behavior independent of inner experiences (Skinner 1971; Wolpe 1958), existential approaches to psychotherapy are rooted in phenomenology which stresses the importance of “first-person” accounts of experience. Historically, this difference has led to quite a few disputes between these two traditions of psychotherapy. Especially early behavioral approaches (Skinner 1971) tended to dismiss the “first-person” perspective that is central in phenomenology and existential phenomenology (Van Deurzen 2010) as unscientific or pre-scientific. In spite of these differences, there were early attempts to reconcile these two perspectives (Ryback 1972).

### Existential Approaches to Psychotherapy

Existentialism is not a philosophy but a mood…This temper can best be described as a reaction against the static, the abstract, the purely rational, the merely irrational, in favour of the dynamic and the concrete, personal involvement and ‘engagement’, action, choice and commitment… (Friedman 1964, p. 3).

It is not easy to define existential approaches and delineate them from other psychotherapy approaches—borrowing Mick Cooper’s (2003) term, there is a “Rich Tapestry of Existential Therapies” (Cooper 2003, p. 1). According to Cooper (2003), all of these diverse traditions share a focus on the “givens” of human existence—facts that cannot be “solved” like problems, but that accompany humans during their lives (e.g., death and responsibility). Such “givens,” while at times disturbing, challenging, and anxiety provoking, are fundamental to what it means to live a human life. Forms of “psychopathology” from an existential perspective are viewed as “problems in living.” Such problems in living are themselves expressive of an individual’s attempts at managing the anxiety and disturbance that arises from existence itself and as such all forms of psychopathology are also “attempts at solution.” Emmy van Deurzen, in another attempt to define existential approaches, argues that “Existential thinking is a steadfast and loyal endeavor to reflect on everyday human reality in order to make sense of it” (Van Deurzen 2010, S. 1).

Existential approaches to psychotherapy are rooted in the European tradition of existential philosophy (Bakewell 2016) and were made available for an American audience during the late 1950s (May et al. 1958). Existential philosophers that have become important for existential psychotherapy include the German tradition of (existential) phenomenology (Edmund Husserl, Martin Heidegger; Husserl, 1970; Heidegger, 1927/1962) as well as the French existentialists (Albert Camus, Jean-Paul Sartre, Simone de Beauvoir, Maurice Merleau-Ponty; Bakewell, 2016; Noyon and Heidenreich, 2012). Karl Jaspers, a German psychiatrist who introduced phenomenology to psychiatry (Jaspers, 1932/1969, 1913/1997), was also an existential philosopher. Existential psychotherapy encompasses works by Ludwig Binswanger (1942), Medard Boss (1979), Irvin Yalom (1980), Rollo May (1983), Emmy Van Deurzen (2010), Hans Cohn (1997), and Ernesto Spinelli (2015). Viktor Frankl, an Austrian psychiatrist and the founder of logotherapy, also became very well known in the USA with his work. He achieved this not only with his technical contributions (e.g., Frankl, 1946/1963) but also especially with his autobiographical report on his experiences in death camps (Frankl, 1961).

#### Yalom’s Approach to Existential Psychotherapy

The work of Yalom (1980) has been particularly influential and he is widely regarded as being the foremost “existential therapist.” Yalom, however, regards existential therapy as not itself constituting a specific school of psychological therapy but rather as an attitude and a stance that can be integrated with a wide range of therapeutic approaches and this would certainly include CBT. This attitude is centrally concerned with an awareness of the basic aspects of living and a sense that the therapist is in many respects “in the same boat” as the client. For Yalom, the therapist’s existential attitude and willingness to engage the client in a direct and deeply human manner in a collaborative exploration of the dilemmas of living, in contrast to an expert led attempt to “cure,” educate, or change the client, is what gives therapeutic encounters their effective quality.

Yalom (1980), based on his reading of the European existential philosophers and therapists, identified what he regards as the basic “existential givens” that can be seen to be at play in many forms of psychological suffering and where the key issue is the extent to which a client has managed the task of creating some form of meaningful response to these givens. Often, this “meaningful response” is seen to be an attempt to avoid, cover up or deny the impact of these existential “facts of life.” Yalom (1980) identified four basic existential givens: death, freedom, isolation, and meaninglessness. Each of these givens can be seen to be implicated in a very wide range of psychological problems.

Yet, for many European existential therapists, Yalom’s work does not provide a very accurate representation of the approach and the discussion of the four existential givens seems simplistic. Authors such as Spinelli (2015) have argued that, in fact, the contributions of existential therapy are not primarily to be found with respect to the identification of the four givens of existence. In one sense, there is nothing uniquely “existential” about any discussion of issues surrounding the human experience of death, isolation, meaninglessness, and freedom. It is possible for each of these themes to be meaningfully and usefully explored, and worked with therapeutically, from a wide range of different perspectives.

#### (Existential) Phenomenology

The unique contribution of existential thinking is not limited to the identification of specific “existential themes” but rather in the “manner” in which such themes are explored and clarified philosophically and in therapeutic dialogues. Understanding what this “way” of existential thinking entails involves engaging with the nature of “phenomenology.” Phenomenology is both itself a range of philosophical perspectives and a method for exploring philosophical issues and concrete phenomena in the world. Phenomenology can be defined as the exploration and clarification of “what reveals itself in the way it reveals itself” (Ihde, 1986). A “phenomenon” is thus understood in terms of “appearances” in contrast with what things may or may not “really” be, separate from our human way of engaging with, interpreting, and make use of or responding to things.

There are many different presentations of “the phenomenological method”; however, one of the clearest presentations of this has been provided by Spinelli (2015). Spinelli has argued that the essence of the phenomenological method can be outlined in terms of three overlapping and interdependent phases or steps. A phenomenological investigation of any phenomenon, such as the experience of “being anxious leaving the house,” or “being depressed about social distancing,” or “protesting about the necessity to wear a face mask,” would involve the following:

1. The phenomenological reduction: In the first step the investigator (or therapist) is encouraged to identify and set aside pre-conceptions, personal biases, or theoretically based views regarding the nature of the phenomena under investigation including any ideas regarding the causal factors involved in its generation or maintenance. In some respects, this step is close to a mindful state of “being with what arises in awareness as it presents itself” that will be familiar to practitioners of mindfulness-based approaches as well as the “defused open awareness” described in Acceptance and Commitment Therapy.
2. The rule of description: This step or “rule” can be summarized as “describe, don’t explain.” The phenomenological practitioner is encouraged to attempt a descriptive clarification of the experience or object under investigation—how is “being afraid of leaving the house” experienced? What does it entail? How is this experienced bodily and within the field of meaningful relationships that the individual undergoing it has their existence? Again, these descriptions should be as free of “explanatory hypotheses” as possible.
3. The rule of “horizontalization”: In this final step, the phenomenological practitioner is urged to avoid placing any, potentially biased, “hierarchies of significance” on the experience rich descriptions that were obtained via step 2. Thus, a client’s reports of an increase in heart rate and a concern about potential heart failure when leaving the front door are regarded as being as potentially relevant and “unknown” as to their potential implications as the client’s statements regarding “should I or should I not wear a mask, and what will the neighbors think if I do wear one?” and “actually I enjoy the slight sense of social distance that arises and the freedom this implies when I do wear one.”

Key to understanding the phenomenological method is that in most instances, it can be said to ultimately be a failure. That is, it is never a complete possibility to identify and set aside all of one’s assumptions and theories and most descriptive interpretations of experience can be seen to contain at least some, often hidden, explanatory components and any act of identifying an aspect of the description worthy of further investigation can be seen to be a movement away from horizontalization. It is the effort at increased openness, and the attempt to remain at a primarily descriptive level that is regarded as being fruitful, both for the practitioner themselves and the effort at gaining a greater sense of “understanding.”

Existential philosophers such as Heidegger (1927/1962), Sartre (1943), and Merleau-Ponty (1962) have all utilized the phenomenological method in their investigations to the extent that their philosophies are understood as expressions of existential phenomenology and their treatment of existential themes is inseparable from the manner in which they employed phenomenology. While their work does contain many useful and complex discussions of existential themes such as death, freedom, meaninglessness, and isolation, far more fundamental and consequential to each of their philosophies is their clarification of the fundamental “relational” or “inter-relational” nature of human existence. For Heidegger, human existence is always being-in-the-world as well as being-with-others. The infamous Heideggerian hyphens are there in order to emphasize the inseparability of human existence and the world. This can be seen to contrast with the more “Cartesian” perspective that seeks to separate subject from object, self from other, emotion from cognition. The terms “Cartesian” is of course derived from the work of Rene Descartes, and the work of the existential-phenomenological philosophers is in many respects and attempt to “overcome” the problems inherent in the Cartesian view such as “how exactly does cognition relate to emotion and behavior?”

### (Cognitive) Behavioral Approaches to Psychotherapy

It is beyond the scope of this paper to comprehensively describe the history of CBT (see Rachman, 2015, for a thorough review)—rather, we will focus on the most important developments and we will show that the different traditions that have merged into CBT have quite diverse relationships to existential approaches.

#### Behavioral Traditions

The origin of what today is called CBT lies in the application of learning theory (classical and operant conditioning) to clinical problems such as anxiety (e.g., Wolpe, 1958) and early texts focus on laboratory work with animals such as rats and pigeons and the application of results to human clinical problems. The language of early behavior therapy was full of terms such as “systematic desensitization” (Wolpe, 1958) and “counterconditioning” (Davison, 1968); behavior therapists were described as “social reinforcement machines” (Krasner, 1962). Obviously, the language used in existential approaches differs strongly from these terms and early proponents of existential approaches tended to be highly critical of behavioral approaches (typically called “rat psychology”) while behavior therapists tended to berate existential writers as “unscientific.”

#### Cognitive Therapy and Cognitive Behavior Therapy

As is well known, cognitive approaches gained influence in general psychology (e.g., Miller et al. 1960) and later also in psychotherapy. A.T. Beck’s work stressed the importance of cognitive constructs such as attitudes and schemas in the development and treatment of disorders such as depression (Beck et al. 1979). Similarly, Albert Ellis (1962) stressed the importance of reasoning for psychotherapy. Interestingly, both Beck and Ellis included elements from behavioral traditions into their systems of psychotherapy, and in this way, “Cognitive Behavior Therapy” and “Rational Emotive Behavior Therapy” were created (Ellis, 1995). Even though Beck still spoke of “Cognitive Therapy” in the early 1990s (Beck, 1993), there was a clear trend to merge these two traditions (Rachman, 2015, p. 5: “The adoption of cognitive concepts into therapy was inspired by the development of cognitive psychology and by a desire among clinicians to pay more attention to the humanistic concerns of their patients. Behaviorism left little place for the content of the patient’s anxieties, and clinical conversations and analyses were regarded as distractions from the need for patients to *wend* their fearful way up the anxiety hierarchies.”)

#### Modern (Third Wave) Approaches in CBT

Current approaches in CBT have expanded the theoretical basis enormously and terms such as mindfulness (Segal et al. 2013), acceptance (Linehan, 1993), values (Hayes et al. 2012), and compassion (Gilbert, 2010) have entered the terminology of CBT. Even though the term “third wave” is controversial, Hayes’s 2004 statement nicely summarizes these developments:

The third wave reformulates and synthesizes previous generations of behavioral and cognitive therapy and carries them forward into questions, issues, and domains previously addressed primarily by other traditions, in hopes of improving both understanding and outcomes (Hayes, 2004, p. 658).

### CBT and Existential Approaches

Taking a look at the section on the development of CBT above suggests that there can be no simple answer to the question “what is the relationship between CBT and existential approaches?” Rather, each of the traditions above might give a different answer: from an early learning theory perspective, existential approaches were lightyears away, but the work of Albert Ellis and Aaron T. Beck incorporated ideas that were at least compatible with existential issues. Modern “third wave” approaches with their emphasis on values and the like may sometimes sound like existential writing—yet, very often the more subtle notes of existential phenomenology are lost.

Cognitive behavioral and existential approaches to therapy have been noted by numerous authors as being so different in their assumptions and practices as to be representing opposite ends of the psychotherapeutic spectrum (Ottens and Hanna, 1998). Yet, in his seminal work outlining the cognitive model of psychopathology and psychological therapy, Beck acknowledges the influence of the existential and phenomenological philosophies of Heidegger and Husserl as well as the contributions of the phenomenological studies of Jaspers, Binswanger, and Strauss (Beck et al. 1979). More recently, Clark, Beck and Alford (1999) have stated that the philosophical perspective that most clearly captures the insights and concerns of cognitive therapy is existential phenomenology. Moss (1992) has argued that Beck and other cognitive therapists have effectively smuggled central insights from existential phenomenology into the respectable halls of academia via the “back door.”

However, cognitive behavioral therapy was never presented as being principally a working out, or direct application, of existential phenomenology, and there remain a number of significant tensions and inconsistencies that have implications for the practice of therapy and may constitute important issues for dialogue and debate: CBT from an existential perspective is expressive of a Cartesian point of view especially when it is maintained that the essence of psychological problems lies in the individual’s possession of internal and distorted “cognitive representations” that the therapist is in a position to help identify and correct so that they accord more adequately with the way the world really is. This fundamental idea that psychological health will be promoted by a more accurate “correspondence” between internal cognitive representation and external reality is deeply challenged in existential thinking. In CBT, this “correspondence theory of truth” can be seen to be expressed strongly in the more heavily rationalist cognitive versions of CBT and challenged in contextual behavioral science. This is not at all to deny the reality of cognitive biases and processes of interpretation. In fact, phenomenology is in significant agreement with the CBT contention that ours is an “interpreted reality” (Spinelli, 2015). It is of course possible to be “in error”—I may have a clear memory of leaving my copy of “The interpretation of Dreams” on the dining room table only to later discover that it is in fact a manual for the CBT treatment of panic attacks.

After having set the stage for an encounter between existential phenomenology and CBT, in the next section, we will explore the role of death in psychopathology and we will discuss possible CBT approaches in dealing with death anxiety.

## Death, Psychopathology, and Its Treatment in CBT

Of all the existential issues that have been discussed in relation to mental health, death has dominated the literature (Vos, 2018). There are two central reasons for this. First, it is the earliest existential crisis to arise in a human life. Our knowledge of our own impermanence emerges in the first decade of life and presents existential challenges in children as young as 3 years of age (Hoffman et al. 2010; Menzies and Menzies, 2019). Second, death is a reality that never leaves us. One can find meaning, solidify identity, exercise freedom, and choose purposeful activities and pursuits, but we must forever live in the shadow of the truth of our inevitable death (Yalom, 2008). It is for this reason that William James described death as “the worm at the core” of human existence (1902/1985, p. 119). In the existential tradition, the terms “fear” and “anxiety” delineate different concepts: while fear relates to a reaction to a specific danger or stimulus (such as height), anxiety encompasses a more diffuse state characterized by uncertainty and helplessness (see e.g., May, 1977).

### Terror Management Theory

The most comprehensive and well-researched account of the operation of death anxiety in everyday life is provided by Terror Management Theory (Greenberg et al. 1986). This theory, building on the work of anthropologist Ernest Becker, proposes that the awareness of death has the capacity to cause crippling fear that would make it impossible to function without a range of cognitive defenses. Each of the proposed defenses amounts to little more than elaborate forms of denial.

First, by adopting one of many religious systems on offer, the individual can claim a literal immortality by believing in an afterlife. Menzies and Menzies (in preparation) note that religions without an afterlife of some sort (including reincarnation) are few and far between. As science has answered some of the mysteries of the universe, one might think that religious belief is no longer relevant. On the contrary, more than half of the world’s population still maintain strong religious affiliations (Pew Research Centre, 2012). Pyszczynski and Thompson (2018) argue that fear of death drove the development of religious belief as a balm against impermanence. Of course, these authors are not the first to suggest this. The Roman philosopher Caecilius Statius said essentially the same in 92 AD when he suggested that “fear made the first gods in the world” (Statius, 92CE/2003, line 661).

Second, rather than claiming a literal immortality, one can seek symbolic immortality by adhering to cultural belief systems that provide a purpose or meaning to everyday activities. By buying into cultural structures that were here before you were born, and shall remain after you die, you become part of a larger assembly that is ongoing. This could include supporting a movement (e.g., environmentalism), a nation or state (e.g., nationalism), or even a political party or sporting team. For example, football followers of Liverpool F.C, a club that was founded in 1892, “never walk alone” and die in the knowledge that they were part of a continuing movement.

Greenberg, Pyszczynski and Solomon (1986) argue that heightened self-esteem comes from excelling within the value system of your chosen culture. In other words, self-esteem is built by achieving within the cultural perspective that one adopts. It allows the individual to believe that they have “lived a good life,” making death an easier pill to swallow. If one buys into the subculture of academia, for example, successful promotion through the ranks to Professor will build self-worth, an effective balm against the finality of death. Heightened self-esteem has been shown to reduce death anxiety in several studies (Helm et al. 2018).

Different laboratory paradigms have been used to explore Terror Management Theory. The most popular has been the mortality salience design in which one group receives a reminder of death, typically buried in a long battery of questionnaires to disguise the purpose of the study, and a second (control group) does not. Across hundreds of studies, it has been shown that reminders of death lead to aggression against outgroups, religious intolerance, racism, increased consumerism, greed, desire for more children, and adherence to political identification. Heightened self-esteem, as predicted, has been shown to moderate these effects (see Pyszczynski et al. 2015, for a review of terror management findings). Although results have been mostly consistent, it should be noted that in the context of the replication crisis in psychological research, there were also some mixed findings, e.g., the Many Labs 4 study (e.g., https://psyarxiv.com/cb9er/). As in other areas of psychology, future research on TMT should focus on replication and work with adequate sample sizes.

### Death Anxiety and Psychopathology

If anxiety about death is involved in mediating a broad range of normal behaviors, could it also be a driving force in abnormal behaviors? Iverach, Menzies and Menzies (2014) argue that, on the face of it, many behaviors that we see in mental health disorders seem derivatives of death anxiety. In the phobias, for example, most feared stimuli can be said to increase the chance of death. Fears of spiders, snakes, heights, water, enclosed spaces, planes, cars, and many other situations and activities seem to have death as their logical feared outcome. In illness anxiety disorder, many authors have highlighted the possibility that these individuals show heightened fears of death (e.g., Furer and Walker, 2008; Menzies et al. 2019). Again, the behaviors that sufferers engage in seem linked to preventing death (e.g., increased doctor visits, requests for repeated medical tests, palpating lymph nodes, and self-examination of skin). In panic disorder, individuals often report that they fear having a heart attack or that they will not get sufficient air to continue breathing (Iverach et al. 2014). In OCD, checking of electric outlets, gas stoves, washing, and cleaning behaviors all seem motivated to prevent tragic outcomes that could cause death (Menzies et al. 2015). The same claims could be made for a range of other disorders including somatic symptom disorder, post-traumatic stress disorder, and separation anxiety disorder (see Iverach et al. 2014).

Given these suggestions, Menzies, Sharpe and Dar-Nimrod (2019) examined the relationship between death anxiety and a range of markers of general mental health in a large sample of treatment seeking participants with at least one mental health disorder confirmed on the ADIS-5L (anxiety and related disorders interview schedule for DSM 5–Lifetime version; Brown and Barlow, 2014). Two hundred participants with a range of mental health problems completed a battery of measures, including death anxiety questionnaires, at a large private practice in Sydney, Australia. Twenty-one different disorders were represented in the sample, the most common of which were obsessive-compulsive disorder, generalized anxiety disorder, major depressive disorder, social anxiety disorder, and panic disorder. In this diverse sample, death anxiety was shown to be positively correlated with the number of lifetime diagnoses, total number of medications taken, number of hospitalizations, level of distress/impairment on the ADIS-5L, and the depression, anxiety, and stress scales of the DASS-21 (Lovibond and Lovibond, 1995). Importantly, these relationships were not accounted for by neuroticism scale scores. Similarly, meaning in life and attachment style did not moderate the relationships between death anxiety and markers of mental health. The findings were interpreted as providing consistent support for the claim that death may be the central existential issue that underpins mental health conditions (Menzies et al. 2019).

Of course, correlational studies cannot establish causation. Few studies to date have experimentally tested the hypothesis that death anxiety may be mediating mental health symptoms. Two studies have used a mortality salience design with clinical samples. The first was conducted by Menzies and Dar-Nimrod (2017) and used a large sample of treatment seeking individuals with OCD. The study sought to explore whether reminders of death would preferentially increase cleaning behaviors among OCD washers compared to OCD non-washers (the latter group consisting of individual with checking behaviors, sexual or aggressive obsessions). One hundred and thirty-two participants (66 washers and 66 non-washers) were randomly allocated to either a death priming condition or a control condition. Following priming, participants completed a series of distraction tasks. Most importantly, one task involved completing time pressured tasks while having skin conductance measured. This task was used for the sole purpose of providing the researchers with an excuse to put conductive gel on the fingers of participants, giving them a reason to wash up at the end of the study. Washing behavior was monitored surreptitiously. As hypothesized, death priming was associated with dramatic increases in cleaning efforts (as measured by washing duration, soap and paper towel use) among OCD washers, but not non-washers. In a follow-up study, Menzies, Sharpe and Dar-Nimrod (submitted) have shown that reminders of death increase anxious behavior (i.e., time spent scanning one’s body, identification with images consistent with poorer health, and intention to visit a medical practitioner) among individuals with body scanning-related disorders (i.e., panic disorder, illness anxiety, or somatic symptom disorder), but not a non-scanning disorder (i.e., depression).

In sum, there is now a growing body of findings that suggest that death anxiety may be a critical driver of many forms of psychopathology. This has led to calls for the development of transdiagnostic treatments for death anxiety to supplement standard care.

### Treatment

One of the challenges in the treatment of death anxiety is that death is an existential given. In general, expectancy models of anxiety have emphasized that anxiety disorders arise from overpredicting threat. In the specific phobias, for example, the individual may overestimate the likelihood of being mauled by a dog, drowning in the swimming pool, dying in the plane, and so on. This cognitive error can be corrected through restructuring or exposure-based programs. However, the fear of death itself does not involve a cognitive error. The individual is correct in assuming that, 1 day, they shall not exist. If this is the basis of their fear, one might question whether cognitive behavioral interventions are appropriate and whether psychosocial treatments could offer these individuals anything.

Given this, Menzies, Zuccala, Sharpe and Dar-Nimrod (2018) conducted a systematic review and meta-analysis on psychosocial treatments for death anxiety. In an encouraging finding, results from 15 randomized controlled trials suggested that these treatments produced significant reductions in death anxiety, with a small to medium effect size (*g* = .45). Notably, therapy type was a significant moderator of treatment efficacy (*g* = − 1.39). Cognitive behavior therapy (CBT) was found to be the most efficacious, producing significant reductions in death anxiety relative to control (*g* = 1.7), whereas other therapies did not (*g* = .20). The treatment procedures varied dramatically across CBT programs but typically included exposure (e.g., writing your last will and testament, reading obituaries, resting in coffins, visualizing your dead and decaying body, watching cremations) and restructuring of maladaptive thoughts and beliefs (e.g., “my children’s lives would be ruined if I died,” “death will be a painful experience”). However, it should be noted that the meta-analysis by Menzies et al. (2018) cautions that the quality of the evidence for the included studies was judged to be low and that studies with higher methodological quality are required. More work is needed to establish the CBT procedures that produce the greatest reductions in death anxiety (see Menzies, 2018, for a review of CBT approaches). Further, more research is needed to identify the mechanism of action of behavioral and cognitive procedures in reducing death anxiety. For example, while exposure to spiders reduces phobic estimates of being bitten, it is unclear why exposure to coffins or cremations would reduce the threat of these stimuli.

## Existential Challenges for CBT Training

Despite the historical relationship and indebtedness of CBT to existential phenomenology described above, the education of CBT therapists, at least in the UK, Australia, and Germany, has traditionally not included any direct engagement with existential philosophy or existential psychology.

### Current Psychotherapy Training

Increasingly, with the advent of the Improving Access to Psychological Therapies Programme in England (IAPT), CBT training has been characterized by a focus on disorder-specific models with an associated concern for ensuring adherence and minimizing therapist “drift.” In many respects, the IAPT programme in England has been a remarkable success and has resulted in an increased demand for CBT training and an increased demand for suitable applicants for such training. This has in turn resulted in a significant change in the type of individual receiving advanced CBT training. In years gone by, CBT training may be gained as part of a longer professional, often doctoral level training in which the trainee will be exposed to a wide range of competing and contrasting theoretical orientations. This is still true in many parts of the world. However, in more recent developments, individuals may be accepted onto CBT training, at an advanced level, without necessarily having attained a wide experience of alternative therapeutic perspectives. In their original manual for the conduct of cognitive therapy for depression, Beck, Rush, Shaw and Emery (1979, p. 25) stated “The aspiring *cognitive therapist* must first be, a good psychotherapist.” While this is often the case, at present, it is also the case that frequently individuals enter training without having clearly developed capacities as psychotherapists. For example, many CBT trainees enter training with a background as a “psychological wellbeing practitioner,” a new professional grouping that has been distinguished by it “not being a counsellor or psychological therapist” but rather offering more clearly limited forms of psychoeducation and advice and in which supervision is at least partly focused on preventing a drift by the practitioner into conversations that would be understood as forms of psychotherapy or counseling. The development of this professional group as well as the expansion of provision and training in CBT in the UK has not gone without its critics. Indeed, there have been a range of authors, from alternative therapeutic perspectives, who have lamented what they regard as “The CBT Tsunami” (Dalal, 2018). Others have suggested that the emphasis on evidence-based interventions in IAPT risks the possibility of “100 years of psychotherapeutic knowledge being lost” (Lees, 2016). Such catastrophic predictions are, in our view, evidence of significant cognitive bias and open to challenge. CBT trainees who have a background as psychological well-being practitioners often go on to become highly competent CBT therapists; however, in doing so, there is a degree of “unlearning” required as well as a need to become more fully acquainted with the wider range of psychotherapies available, their assumptions, and worldviews, as well as their technical procedures, if only to become a more rounded and informed professional psychological therapist.

### Standard CBT Techniques and Existential Issues

In the practice of standard CBT, it might often be the case that existential issues such as a fear of death or anxiety about meaninglessness may be clarified as a result of the commonly used “downward arrow technique.” Although trainees and inexperienced CBT therapists are encouraged to avoid a heavy handed use of this strategy, in the hands of an experienced therapist, such a strategy may reveal core beliefs and schemas that are directly expressive of existential themes. As Ottens and Hanna (1998) have argued, many schemas that clients present with can be seen as inherently to do with existential issues. The sense that such cognitive structures are somehow “deeper” may add to the sense that CBT has an easy avenue and preexisting strategies to respond to existential themes. This however would be to miss the point of much existential thinking. Existential therapy has itself often been considered as much as “breadth therapy” as it is a “depth therapy.” That is, existential thinking suggests that concerns arising from existence saturate our daily lives and interactions; they are not essentially buried or hidden or requiring specific techniques to identify. As Oscar Wilde wrote in the “Picture of Dorian Grey”: “It is only shallow people who do not judge by appearances. The true mystery of the world is the visible, not the invisible....” (Wilde, 1891/2000, p. 24). Phenomenology suggests that paying greater attention to how experience presents itself often leads to an “unfolding” of existential significance. Sometimes, therapists and patients as well tend to avoid those topics, because that “unfolding” automatically transfers both therapist and patient to relatively unsafe grounds where there are no simple solutions or manualized techniques. A downward arrow is as relevant as a sideways or upward arrow; more relevant however is the capacity to stay still and to stay with experience and not to hastily suggest that reaching for a specified technique will allow the therapist to somehow “take hold of” and “manage” existential concerns. Existence always exceeds what can be formulated.

### New Developments in CBT Training

Over the last 10 years, Michael Worrell and a number of colleagues in the UK have endeavored to provide training input to new CBT trainees in “other modalities of therapy” in order to assist these trainees in developing the necessary foundational skills as psychotherapist that you would expect any well-training individual to possess who is entering CBT training within one of the defined “core professions” (such as clinical or counseling psychology). This training consists of 3 days of lectures and experiential practice with one of these days focusing upon existential approaches to therapy and their philosophical basis. Consistent feedback from over 200 trainees has indicated that this training in aspects of existential thinking is experienced as both valuable and relevant and also at times highly challenging (Worrell et al. 2018). In the space that remains in this section, we will outline our understanding of what trainees have found to be both highly relevant and enriching about existential thinking as well as some of those aspects that are experienced as very challenging and may in fact serve in part to clarify where CBT (broadly defined) and existential phenomenology constitute very different and at times contradictory world views.

### Existential Contributions

Cognitive behavioral therapy can be seen to be characterized by a positive and optimistic view of life. For every disorder, there is a model, a manual, and a set of techniques and indeed a discoverable set of “key cognitions” and/or “cognitive processes” that, when identified and corrected, will lead to the possibility of the cessation of difficulty and the resumption of positive functioning. Existential thinking, by contrast, is best characterized as being expressive of a “tragic view of life” (De Unamuno, 1954). That is, psychological suffering is seen as an inevitable aspect of human existence, as being rooted in, and expressive of, fundamental “givens” that also provide the foundations upon which human existence is able to reveal itself as “meaningful.” This more “tragic” dimension, however, is by no means completely absent from contemporary CBT. The work of Robert Leahy (2015) has, in particular, drawn specifically on existential philosophy and has sought to highlight the more tragic aspects of existence as well as the need for competent CBT therapists to develop the capacity to “stay with” expressions of existential distress rather than attempting to “reconstruct” the client’s cognitions and remove such distress. Key to this, according to Leahy, is the CBT therapist’s own awareness of their response to the universal givens of existence. Perhaps though the clearest example of this, more tragic view of life can be found in Acceptance and Commitment therapy where the ultimate goal of therapeutic work is conceptualized as being assisting clients to identify and move towards meaningful life values despite the potential ongoing presence of unwanted psychological or behavioral disturbance (Hayes et al. 2012). Readers may have noticed the “ACT like flavor” of the opening quote!

As Moss (1992) has noted, where existential approaches and CBT most clearly agree and have the potential to mutually enrich each other is the central place both perspectives give to the issue of “meaning.” The strength of CBT can be seen to arise from the highly detailed analyses that it has conducted regarding different forms of meaning, and processes surrounding the construction of meaning and its maintenance and change. Existential approaches can be seen to provide a much-needed contribution through its emphasis on much “broader” or indeed “deeper” forms of meaning that relate to the issue of “what is a meaningful life?”

Existential approaches to therapy, drawing upon their philosophical roots, are also inclined to emphasize notions of embodiment as well as regarding emotional experience as a fundamental aspect of human existence that cannot be adequately understood as primarily the end result of cognitive processing. Existential anxiety, for example, is not something that is seen by existential therapists as a target for “elimination.” While a specific “fear of death” might be a legitimate target for CBT and, as reviewed above, may well be seen to reduce as a result of interventions like exposure, for the existentialists, this would by no means be an indication that existential anxiety has been “overcome.” As a fundamental aspect of existence, existential anxiety is an aspect of what it means to be human. We “are” anxiety in a sense. In existential philosophy, an important distinction has been drawn between the “ontological” and the “ontic” (Cohn, 1997). For something to be regarded as ontological, it is being suggested that this is a fundamental aspect of “Being” of existence itself. For something to be considered ontic, on the other hand, indicates that it is regarded as a specific contextually derived instance of some phenomenon. Existential anxiety is regarded as ontological; it is fundamental and constitutive of human existence. Even where I do not psychologically experience anxiety, I am nevertheless existentially anxious. Our attempts to finish this paper, to express our excitement and interest in the possibilities of a dialogue between CBT and existential philosophy, is no less expressive of existential anxiety than any phobic avoidance of an object such as a spider, it is simply that this ontological aspect is not, for the most part, clarified.

CBT trainees, while often struggling with the abstract nature of some of these ideas, which are in some respects alien to CBT thinking, nevertheless often appear to find an emphasis on the embodied aspect of emotional experience very helpful. That is, taking a more descriptive focus on how the client experiences a negative emotion at a lived embodied level allows a much great and clearer access to what these emotions mean to the client. Experienced CBT therapists of course know this, as the easiest way to get lost in an exploration of a client’s cognitions is to fail to link this clearly to how such cognitions are an abstracted part of what is in fact an experiential flow of “embodied-emotion-meaning-action.”

Similarly, Existential philosophy has suggested that human beings are fundamentally “guilty.” This sounds like a moral or religious pronouncement; however, it is not. Rather guilt as ontological, or existential guilt, indicates that human beings “always already” lag behind their “possibilities for being.” This can be expressed ontically in both life-affirming as well as life-disabling ways. The urgency that is felt by the present authors to complete the paper and the concern that important aspects will be inevitably missed or expressed badly or will be misunderstood may function to motivate us to redouble our efforts and to push each other towards greater clarity. On the other hand, the client suffering from OCD, who is concerned that, by their actions or inactions, have “infected” others with germs is also expressing their difficulties responding to the given of existential guilt. The point here is that it is a mistake to see existential givens as equivalent to some form of psychopathological process that can be corrected by the right intervention. As Sartre maintains, there really is “no way out.” Of course, therapeutic interventions may be successful in assisting an individual to take up a more creative stance towards the existential givens and one that allows them to “widen their world” rather than narrowing it is a futile effort to eliminate all uncertainty, all existential guilt, and all existential anxiety.

A final mention must also surely be made of the issue of “Time.” Heidegger’s master work was of course titled “Being and Time” (1927/1962). Again, as a fundamental aspect of existence, time can be regarded as constitutive of human existence. Just as we are anxious, we *are* time, that is, human beings “live time.” This can be contrasted with what might be called “clock time.” Clock time is measurable and constant, it is precise and predictable. Lived time on the other hand is not. The client who has presented with depression, for example, may experience the 50-min session as an impossible stretch of time. It seems vast as well as futile. The CBT emphasis on providing structure and frequent summaries is at least in part an attempt to provide some much-needed support to a client who experiences the flow of time in such a fashion. By contrast, a client who is experiencing general anxiety may find the session unacceptably and unaccountably “too quick,” and again, the structural aspects of CBT may assist such a client in slowing down and registering what progress is in fact being made. Beck’s “cognitive triad” includes explicitly the dimension of time via the issue of the future. Existentially, however, this needs to be seen in a more complex fashion. Existential time is also understood as being “multidimensional,” that is, the “past” is always seen as being an aspect of the present moment and this present moment as always being “future directed.” This leads to some rather startling reflections on issues such as taking an assessment. Existential therapist Hans Cohn (1997) for example suggests that the existential perspective challenges the notion of “taking a history” as “there is no history as such.” The way in which a client relates their history will reflect the present moment and their future projects as well as the unique manner in which the past has been carried forward and is always “present.” Cohn suggests that the story the client tells a particular therapist is always the story told to that particular therapist; they may tell a different therapist quite a different story leading to quite a different “formulation.” CBT therapists tend to emphasize that CBT is distinct in emphasizing the importance of working with the present; the existential perspective both affirms and significantly complicates this view.

### Yalom’s Existential Givens and CBT Trainees

As mentioned above, Yalom (1980) identified 4 basic existential givens: death, freedom, isolation, and meaninglessness. In discussions with CBT trainees, it is often the case that they are easily able to identify these aspects in the concerns and dilemmas that their clients have brought to them, regardless of whatever “disorder-specific model” they may be using. Doing so, as well as identifying their own stance towards these four givens of existence, and when their awareness of these givens has at times been experienced as anxiety provoking or disturbing, is reported to contribute to a greater sense of acceptance, compassion, and “fellow feeling” towards their clients. In the current context of the world corona virus pandemic, these four issues are seen as very much an aspect of our shared experience of living. While a consideration of the four existential givens may be seen as highly “philosophical” for some, the relevance of this for CBT is strengthened considerably by a consideration of the empirical work that has been undertaken on the relevance of these issues. As noted above, by far, the greatest amount of work has been conducted on the issues of “death awareness” and “death anxiety” under the aptly named “Terror Management Theory” (Greenberg et al. 1986). The experimental basis of this work provides fertile ground for CBT therapists to start exploring the potential relevance of these findings to their work with clients (see further Iverach et al. 2014). In summary, CBT trainees in their encounter with existential thinking often find that the emphasis on “meaning,” with this broader and deeper sense, as well as upon the fundamental aspects of human existence, as this has been discussed and elaborated in existential approaches to therapy, appears consistent with and potentially enriching and enhancing of CBT practice.

### Experiential Experiments for CBT Trainees

The reader of the sections above may well now have an embodied experience similar to that of many CBT trainees when first exposed to these more complex forms of existential thinking—that is, a headache and a desire to move onto something more obviously practical. Part of the difficulty lies certainly with the language used by existential philosophers that often makes even relational frame theory seem like the clearest and friendliest possible presentation of a concept ever rendered in English. At this point in the training provided to CBT trainees therefore, it is usually helpful to present a range of very simple, yet experientially challenging exercises designed to give trainees a “taste” of what it might be like to work from a more existential-phenomenologically informed perspective. Two of these are briefly presented below. In each case, it is important to emphasize that the exercise is not presented as a task in which the trainee is attempting to demonstrate a therapeutic competence in the way in which it should be used in the practice of CBT; these are experiential exercises that are not intended to lead to a “correct awareness” or an “improved performance.” Like an experiential exercise introduced to a client in ACT, whatever the trainee experiences, including an unwillingness to stay with the exercise, is taken as a meaningful response worthy of further reflection.

### Exercises in Existential-Phenomenological Listening

These exercises are conducted in groups of three; there are three “positions” of “therapist,” “client,” and “observer.” Each individual should have time to engage with each of these positions in the course of a practice session. In each exercise, the “client” is asked to describe a recent experience where they had some sort of emotional response to something; this can be positive or negative. The task of the client is simply to describe their experience to the therapist. The exercises then place different requirements on the “therapist”:

- Exercise 1: In the first exercise, the therapist is asked to “simply listen.” They are asked to attempt to “connect or attune to” the client by their bodily felt experience; however, they are instructed not to ask any questions or make any verbal statements at all. At the same time, they are instructed to “listen to yourself attempting to listen to another.”
- Exercise 2. In the second exercise, the client again describes an experience, and this time, the therapist is allowed to speak; however, all their statements must be devoted to the attempt at facilitating the client’s description of their bodily experienced emotions. Such requests for further clarification and description should be minimal and brief. There should be no attempt to link emotions to cognitions or to provide problem solving.

In each of these exercises, normal “self-care” rules apply, and each participant is reminded that they need only reveal aspects of their experience that they wish to, and that they can stop at any times. At the end of each exercise, which in most instances lasts no more than 10 min in each role, participants are asked to feedback to each other and the larger group they experience of what this was like. Feedback over many years reveals a wide range of experiences. Participants find the experience of “just listening” and “attuning to bodily felt emotions” anxiety provoking, freeing, invigorating, annoying, informative, therapeutic, and a waste of time. Each of these reactions can then be discussed in terms of the participants’ assumptions regarding “what it means to be a CBT therapist.” Again, it is emphasized that each of these exercises is artificial and not intended as a practice exercise to develop specific CBT competencies but rather as one way to get an embodied sense of what the existentialists may be talking about. The most common reaction appears to be, “there seems to be something to this worth thinking more about.”

In summary, we believe that the training of CBT practitioners may be substantially enriched via an engagement with existential thinking. This includes both an engagement with the ideas of the existential philosophers and how these ideas have been taken forward by a wide range of different existential therapists, as well as via experiential exercises that are designed to foreground the challenges of more phenomenological ways of engaging with experience. The extent to which such training can be said to improve, enrich, or indeed get in the way of the development of specific CBT intervention competencies is of course an unanswered empirical question.

## The Role of Existential Issues for CBT Supervision and Personal Development of CBT Therapists

Doubtlessly, supervision is a very important part of CBT training, maybe even the most important element (e.g., Alfonsson et al. 2020; Alfonsson et al. 2018; Bennett-Levy et al. 2009). There are different purposes that are served by CBT supervision, and of course, the content-related aspects, e.g., knowledge about specific disorders and treatment ideas, play a crucial role. But we are convinced that besides these “clinical” aspects, there are other issues that could and should be addressed in CBT supervision, and personal development is one of the most important ones. Especially in this regard, existential thinking has a lot to offer in our opinion, and we want to point out how supervisors can help supervisees develop a broader personal therapeutic stance by addressing existential aspects not only when talking about the patients in treatment but also of the therapists in training themselves.

### Supervision in Different Areas of CBT

One of the strengths of CBT is that for almost every mental problem, there is a treatment manual that can be used as a guideline towards best practice. During their psychotherapy training, CBT beginners regularly learn that they should stick to the manual in order to deliver the best available therapy and thereby maximize therapy outcome. Especially, therapists in training who work in university outpatient clinics and participate in associated research projects with patients who meet very specific inclusion criteria often learn to expect more or less “uniform” patients with symptoms that can be addressed in a manualized way. In “mainstream” CBT, trainees are expected to focus on the symptoms at hand and show strong adherence.

Even if manualized therapy is not uncriticized, there is almost no doubt that manuals play a very important role in CBT training and practice (Milne, 2016). But it should be added that in the past years, new ideas of thinking about psychotherapy have emerged: while “classic” manuals focus mainly on disorder-specific knowledge and derive treatment ideas tailored to the symptoms of a specific disorder, on the other hand, “process-based CBT” has been developed (e.g., Hayes and Hofmann 2018). According to this relatively new perspective on CBT, the focus lies on basic principles behind specific interventions and thus represents a transdiagnostic viewpoint. In supervision, a process-based point of view seems of very special interest to us because supervisees do not only need to know and learn how to treat a specific disorder but also to get a deeper understanding of how therapy works on a basic level and which principles are helpful in relation to working with various patients. Or to put it more bluntly: in order to become a “good therapist,” beyond knowing about specific disorders and treatment techniques, one ought not only to have insight into knowledge of specific disorders and treatment techniques but also to have insight into how “things in the world” are working at all. It might also be said that in order to be a good therapist, one needs to be “wise,” at least to a certain degree, and therapeutic wisdom seems to be especially linked to the ability to understand and explain common processes of functioning in the world and that “functioning” encompasses much more than just symptoms of clinical disorders (Levitt and Piazza-Bonin, 2016, 2017). We think that existential thinking can help supervisors foster the development of “therapeutic wisdom” in young trainees. To do so, they need to broaden their own horizon beyond symptom-specific knowledge and allow themselves to integrate insight on life itself into the supervision process.

### Learning about “Real Life”

When therapists in training are confronted with “real-life patients,” they are often confused because of the multitude of issues that their patients have to deal with and that they want to receive help for. It becomes especially interesting when they meet patients who do not have “simple” symptoms or disorders that can be categorized according to the systems of DSM-5 or ICD-10 but that are linked to existential issues such as the meaning of life, death and dying, guilt, responsibility, isolation, and freedom. What happens quite often in supervision is that therapists in training feel helpless and insufficient when confronted not with “problems” that they can help to solve, but with “facts” that are existential aspects of life and that cannot be solved but just be confronted in a more or less useful way. It can be very unsettling for therapists (especially beginners, but to also “older” therapists) to sort out the different role aspects that come with therapeutic work. Of course, this is well known within therapeutic fields where existential issues are very prominent from the very beginning, e.g., psychotherapy of cancer patients (e.g., Breitbart et al. 2018). But during our own work as CBT supervisors, we experience that analogous situations also arise in the work with patients with less severe issues. This derives from the fact that existential issues really are “everyday life,” and therefore every human being is confronted with existential questions sooner rather than later. Of course, not always do those questions enter the therapeutic space, but in our opinion, therapists always need to be prepared to address (or to welcome) such issues. Therefore, they need to be not too afraid of all the questions that might arise in the ubiquitous existential circumstances patients may present, and especially to be not too afraid about not having all the answers (if any) to the questions that patients may struggle with. The massive success of the so-called “third wave” of CBT might be explained by the fact that the respective approaches—e.g., Acceptance and Commitment Therapy (Hayes et al. 2012) Mindfulness-Based Cognitive Therapy (Segal et al. 2013), and Compassion-Focused Therapy (Gilbert, 2010)—all focus heavily on unchangeable aspects of live, that is, on “facts” that cannot be changed fundamentally but have to be addressed in a different manner.

Psychotherapists have to be prepared to deliver both solution-focused attention when problems are definable and stance-altering interventions when existential issues are displaying themselves. The idea of pointing out completely different attitudes in dealing with critical situations is of course not new. For example, 30 years ago, Brandtstädter and Renner (1990) described two generally different coping modes: (a) assimilative coping strategies, where developmental circumstances are transformed in accordance with personal preferences, and (b) accommodative coping strategies, where personal preferences are adjusted according to situational constraints. Also, it could be concluded that the differentiation between “acceptance” and “commitment” in ACT (Hayes et al. 2012) points in the same direction. Is often the case—“true” ideas are very seldom owned by just one therapeutic perspective but are normally an ingredient of different approaches. Both psychotherapy and life itself on a big scale are characterized by confronting the therapist (and the human being) with dilemmas—therefore, understanding the nature of a dilemma and thereby avoiding to try to solve it is one of the key aspects of therapeutic wisdom (Råbu and McLeod, 2018). Supervisors can and should help their supervisees in multiple ways to develop that idea in an increasingly differentiated way over the course of their psychotherapy training, i.e., by reflecting on the existential aspects of life itself, by self-disclosure, and by sharing their own struggle with existential questions and their development over time (e.g., Clevinger et al. (2019), who point out the importance of the supervisor’s self-disclosure), and—maybe most simple—by being a good model in admitting to not having all the answers to all these questions, and to do so without breaking eye contact and doing it hastily while trying to change the subject as soon as possible (“Yeah, death really is a hard issue, but let’s focus on how to prepare that confrontation session properly, ok?”).

### Existential Aspects in the Therapeutic Stance

In supervision, we—as supervisors—have to help younger therapists develop a broad understanding of the different layers of criteria defining our job. On the outer layer, the “classic” CBT training and supervision of sticking to the manual and using RCT results as guidelines for therapy definitely do work—countless studies and meta-analyses have shown that CBT works for almost every disorder. But the “deeper” one gets into contact with the inner fears, hopes, dilemmas, ambivalences, … of a patient, the less useful a problem solving attitude in therapy normally becomes. We need to prepare therapists in training for the fact that, sooner or later, they will face patients who struggle with existential aspects of life that are not a “mental disorder” but may need therapeutic support nonetheless. It is crucial to help younger therapists accept these challenges in therapy without doubting themselves substantially (“As a therapist I am totally insufficient and misplaced if I do not immediately have the easy and clearly working solution for existential questions of my patients”). In developing a therapeutic stance, we think that supervision is the most important part of psychotherapy training (often in combination with self-experience; Bennett-Levy, 2019). Bringing together CBT and existential psychotherapy in supervision helps to develop that therapeutic stance towards a differentiated view that places psychotherapy on a continuum that spans between “manual-oriented psychoeducation and problem solving” on the one hand and “supporting patients in their struggle with life” on the other.

The most important way to do this is to invite the therapists to confront their own existential issues and reflect on their meaning for their own lives. This is where supervision and self-experience merge and—by fostering personal growth—help the therapist develop the ability to decide which part of his or her therapeutic persona is appropriate in which therapeutic situation. Normally, this goes hand in hand with an increased willingness of the therapist in training to show self-disclosure in therapy—and there is solid empirical evidence that self-disclosure is very important in psychotherapy (e.g., Miller and McNaught, 2018; Ziv-Beiman et al. 2017). All the issues mentioned above are very important aspects of the nature of the therapeutic relationship with the patient, and we consider the quality of the relationship to be of paramount relevance for the treatment of all patients, but especially patients with existential issues. Patients are not primarily carriers of symptoms but human beings who deal with their lives in all their light and dark sides on a daily basis. Carefree moments in which existential questions play no (conscious) role at all can pass almost seamlessly into existentially confronting situations. To put it another way: the existential can wait around every corner; however, harmless and everyday it may seem. The therapeutic task is to support people in making the best of life’s paradoxes and to be fully convinced of this possibility. In art, this seems to the author to be expressed more frequently, more honestly, and often more powerfully than in science. A line that has become famous from Leonhard Cohen’s “Anthem” reads: “There is a crack in everything. That’s how the light gets in.” To stick to the conviction of light behind the crack is one of a therapist’s most important tasks, and accordingly, supervisors have to prepare those entrusted to them as best as they can.

It is our impression that CBT, at least in its traditional roots, does not have the strongest focus on the aspects described here. For a long time, that seems to have been the domain of particularly psychoanalytic or psychodynamic thinking—accordingly, a vast amount of literature can be found in that area (e.g., Kahr, 2018; Pinto-Coelho et al. 2016). Reflecting with supervisees at the end of their psychotherapy training which sessions or elements of the supervision have been most helpful and important to them, they regularly explain that our sessions on existential aspects have had the greatest impact. We think that the third wave of CBT brings many benefits to our profession. Of course, this is a relatively new development, and accordingly, a lot of research still has to be done on that matter.

CBT and existential therapy may be understood as expressing quite different views of “the image of the human being.” The main contrast is between a CBT image that is really still in terms of “Man as Machine” where there is the possibility of the correction of “faulty” parts even when this is framed in the more sophisticated language of information processing. The existential image is one of “openness to existence” and does not contain the notion of any straightforward “correction” of faulty parts. The existential view is rather that any possible change in a human being’s manner of existence always changes the whole of that being’s “way of existing” and any such change is also unpredictable as to its consequences both negative and positive. This difference is more important than the initially insurmountable differences, for example, between CBT as a natural science approach emphasizing a more deterministic view and existential therapy as promoting the reality of “freedom.” Freedom in existential thinking is as much about situational and other limitations on freedom as it is about possibility and choice. Existential freedom is always “situated” or “thrown” as Heidegger would have it. So, an existential therapist has no difficulty for instance acknowledging the impact of “contingencies of reinforcement” and “learning history” in constraining what may show up as a possible choice.

## Conclusions: Challenges and Potentials of the CBT/Existential Crossroads

This paper described a number of aspects at the crossroads of CBT and existential psychotherapy. We hope that it has become clear that this is a highly interesting perspective that nevertheless has its perils and pitfalls.

Starting with the *challenges* of this encounter, we fear that the incorporation of existential principles and procedures into CBT may fall short of its real potential: even though most “existential” writing circles around the givens of existence, this is merely a starting point for existential thinking and intervention and especially the phenomenological method should not be understood too “shallow.” Phenomenology, when practiced in depth, really does challenge and change the practitioner. It is a demanding practice that requires the practitioner to be ever aware of the ways in which they import assumptions and theories into their encounter with the phenomena of the world, including their experience of clients. The effort to set these aside comes at a cost of greater uncertainty and anxiety. It can be so much simpler to “follow the manual.” Another challenge for CBT (therapists) may be to truly acknowledge the work of existentialist philosophers and psychotherapists: some of these authors are very hard reading (e.g., Heidegger with all his neologisms) and potentially impossible to operationalize. Indeed, for many existential philosophers and therapists, the attempt to “operationalize” existential thinking will inevitably lead to distortion and error. Undoubtedly, the major criticism of existential philosophers and existential therapists is that they are frustratingly “wooly.” Reading Heidegger, for example, is frustrating because he does not set out his arguments in a logical manner, he repeats and restates things, and he uses ordinary words in an unusual and atypical way. In many ways, his writing is more poetic (although not necessarily good poetry), a sustained repetitive meditation on the issue of “Being” or “what does it mean that there is something rather than nothing at all?” This charge of wooly thinking can be equally leveled at many existential therapists who point blank refuse to operationalize or even describe their way of conducting therapy, insisting instead that such things cannot be manualized or even described as each therapeutic encounter is unique. This apparent mysticism is hardly conductive to productive dialogue or the project of contributing to the evolution of psychotherapeutic practice. Reading the case studies of Yalom, for example, it would appear that the only person capable of such work is Yalom himself. If psychotherapeutic input is regarded by existentialists as a “good,” then some significant work needs to be devoted to the issue of how others are to be more effectively trained to work with the existential dimensions of clients’ presentations. Finally, the major challenge for CBT may lie in the fact that existential approaches are systems of psychotherapy in their own right and that an encounter should occur on eye level rather than on a basis to conquer territory.

Besides these challenges, we believe that the crossroads of existential psychotherapy and CBT holds a large *potential*. Following the main arguments of this paper, we believe that existential philosophy and psychotherapy incorporate a large amount of insights that can be highly inspiring for CBT theorists and practitioners: from the role of death anxiety for understanding psychopathology and for designing interventions to reflecting upon existential concerns in the context of training and supervision, there is a huge area that can be explored in more detail during the next years. As spelled out for the role of death anxiety, the other givens of human existence can be put to experimental scrutiny.

In CBT training, didactic presentations of the history and relationship between CBT and existential philosophy and therapy may be part of providing CBT therapists with an appropriate theoretical and philosophical “grounding.” Experiential exercises that support the capacity of trainee therapists to “stay with” distress rather than immediately “leaping in” with interventions and solutions may also be of considerable benefit. Supervision is obviously important in supporting trainees to know when staying with or leaping in is called for and most appropriate. Such distinctions may evade even the most precisely drawn up manual.

One key possibility is to actively teach and model the use of the phenomenological method as an important resource for CBT therapists. The phenomenological method holds great promise as a method to support trainees in learning more adequately how to embody the CBT principle of “guided discovery.” If guided discovery really is being conducted in a spirit of mutual discovery, rather than the therapist leading the client down a pre-determined path, then the phenomenological method, with its emphasis on the importance of “staying with” experience in an unusually challenging and open way, holds great promise both as a training method as well as one of the fundamental and indispensable “competencies” of CBT practice.

In CBT supervision, incorporating elements from existential philosophy and psychotherapy may be very helpful in developing at therapeutic “stance” that allows trainees to constructively work with clients’ existential concerns rather than trying to find “solutions” where there are no solutions to be found. Further, we assume that this existential perspective enriches self-experience of CBT trainees.

At the heart of many existential approaches to psychotherapy is the notion of dialogue or more simply conversation. This notion of the therapeutic relationship as conversation promotes a much greater “collaborative” and egalitarian view. We believe this perspective could be extended to the question of the relationship between existential philosophy and therapy and CBT. Rather than attempting some form of hasty integration, which risks losing the distinct contributions of each tradition, we propose an ongoing dialogue which, like any good conversation, is inherently unpredictable in what it may lead to. Neither party is ultimately “in control” nor in the driving seat in any good conversation. Both parties may be affirmed as well as challenged in their perspectives. Such possibilities include enriching CBT with new strategies and interventions that allow CBT therapists to work with key existential issues (see Menzies, Menzies, and Dingle, in press). This possibility will be no doubt of significant interest and challenge to existentially oriented therapists. In addition, there are many challenges that arise for CBT therapists at both a theoretical and practical “lived” level that arise from a deeper engagement with existential phenomenology (Worrell, in press). We believe that CBT as a whole, and CBT therapists working to be of assistance to other struggling human beings, particularly during times such as the present, where there is a heightened existential awareness and anxiety, will greatly benefit from such an “existential conversation.”
